# Modifiers of short-term effects of ozone on mortality in eastern Massachusetts - A case-crossover analysis at individual level

**DOI:** 10.1186/1476-069X-9-3

**Published:** 2010-01-21

**Authors:** Cizao Ren, Steve Melly, Joel Schwartz

**Affiliations:** 1 Exposure, Epidemiology, and Risk Program, Department of Environmental Health, Harvard School of Public Health, Landmark Center, West, 4th floor, 401 Park Street, Boston, MA 02215, USA

## Abstract

**Background:**

Substantial epidemiological studies demonstrate associations between exposure to ambient ozone and mortality. A few studies simply examine the modification of this ozone effect by individual characteristics and socioeconomic status, but socioeconomic status was usually coded at the city level.

**Methods:**

This study used a case-crossover design to examine whether impacts of ozone on mortality were modified by socioeconomic status coded at the tract or characteristics at an individual level in eastern Massachusetts, US for a period May-September, 1995-2002, with a total of 157,197 non-accident deaths aging 35 years or older. We used moving averages of maximal 8-hour concentrations of ozone monitored at 8 stationary stations as personal exposure.

**Results:**

A 10 ppb increase in the four-day moving average of maximal 8-hour ozone was associated with 1.68% (95% confidence interval (CI): 0.51%, 2.87%), 1.96% (95% CI: -1.83%, 5.90%), 8.28% (95% CI: 0.66%, 16.48%), 0.44% (95% CI: -1.45%, 2.37%), -0.83% (95% CI: -2.94%, 1.32%), -1.09% (95% CI: -4.27%, 2.19%) and 6.5% (95% CI: 1.74%, 11.49%) changes in all natural deaths, respiratory disorders, diabetes, cardiovascular diseases, heart diseases, acute myocardial infarction and stroke, respectively. We did not find any evidence that the associations were significantly modified by socioeconomic status or individual characteristics although small differences of estimates across subpopulations were demonstrated.

**Conclusions:**

Exposure to ozone was associated with specific cause mortality in Eastern Massachusetts during May-September, 1995-2002. There was no evidence that effects of ozone on mortality were significantly modified by socioeconomic status and individual characteristics.

## Background

Substantial epidemiological evidence demonstrates that ambient ozone is associated with daily deaths and hospital admissions [1-5]. These associations are generally consistent across studies or robust to adjustment for secular time trends, short-term variation or weather conditions [6].

Recently, interest in epidemiological studies has grown in how socioeconomic status (SES), individual characteristics (e.g., age, race and gender) and weather conditions modify associations of exposure to air pollution with mortality and hospital admissions. This issue is important in air pollution studies because better knowledge of these modifiers will increase the power to detect risk in future studies and aid in the identification of potential mechanisms of pollutants [7]. Several studies have examined the variation of effects of particulate matter or ozone on health outcomes across temperature levels or seasons [8-13]. Recently, an increasing number of epidemiological studies have examined effect modifications of SES and individual characteristics on associations of particulate matter with health outcomes at population and individual levels [14-20]. However, to date, little work has been directed toward identifying which subpopulations are more susceptible to deaths due to effects of ambient ozone [21]. Many studies have examined effect modification using stratified methods [12,19,20], which will decrease the statistical power and lack the ability to examine directly interaction effect. Moreover, various estimates might be due to differential distributions of covariates across strata, not effect modification because subpopulations were not exchangeable in other variables. A very few studies examined effect modification by socioeconomic status at the city level [1,22]. However, there is more variation in socioeconomic status within urban areas than between urban areas. To date, no study has tried to examine area-based measures of socio-economic status as modifiers at a more geographically resolved area, such as the census tract, or to include both individual and area based measures as effect modifiers.

In the present study, we aimed to examine whether associations between ozone and daily mortality was modified by SES at the census tract level or by individual characteristics using a case-crossover design, which allows for the examination of effect modification at the individual level. As an alternative analytical methodology to Poisson regression, the case-crossover approach allows for the directly modeling of interaction term at an individual level rather than depending on multiple subgroups analyses as in the Poisson design. Consequently, we have the ability to model potential effect modifiers simply using one dataset without rebuilding a dataset when we examined effect modification for different factors. In addition, continuous effect modifiers can be examined without being categorized.

## Methods

### Mortality, weather and pollution data

The study population consisted of 162,146 deceased subjects aged 35 years or older from 3 counties (Suffolk, Middlesex, and Norfolk) in the eastern Massachusetts, USA for the period between January 1, 1995 and December 31, 2002 based on data availability. Residents in the counties consisted of about 44 percent of the state's population. Individual mortality records were obtained from the Massachusetts Department of Public Health, and included all deaths from the chosen counties. The dataset included major characteristics at individual level, such as age, gender, race, education and marital status. Individual residential address was geocoded by a private firm by matching the address to state, city, ZIP code and street network data based on TIGER, and assigning to this street address latitude and longitude coordinates, as well as census tract and block group. The geocoding was then reassessed by us for accuracy and completeness. Using the 2000 US census, we then obtained the population per square kilometer dry land or population density in the block group and socioeconomic status in the census tract, including median household income and percent of the population in the tract with 1999 income below the poverty level at the census tract level.

We identified the following characteristics and SES as potential effect modifiers, age, race, gender, education, marital status, population density, household income and percent poverty. We categorized age into 35-64 years, 65-74 years, 75-84 years, and 85 years or older, education into ≤ 8 years, 9-12 years, 13-16 years and 17 years or more, marital status into never married, married or separated, widowed, and divorced, race into white & non-Hispanic, black, American Indian, Hispanic, Asian or Pacific, and others. We classified population density (residents per km^2 ^in the block group), household income (median household income in the census tract in 1999) and poverty (percent of population in the census tract in 1999 below the poverty level) into low, medium and high using low and high quartiles as cut-offs.

We excluded accident or non-natural deaths in this study (4949, 3.05%) (International Classification of Disease, ICD-9, I-799 or ICD-10 excluding V01-Y98). We classified each case according to their diagnoses based on their primary diagnosis of the following conditions: cardiovascular diseases (CVD) (ICD-9 codes 390-448, ICD-10 codes I00-I79), diabetes (ICD-9 code 250 or ICD-10 code E10-E14) and respiratory disorders (ICD-9 codes 480-486, 490-497, or 507, ICD-10 J100-J118, J120-J189, J209-J499, or J690-J700) [1]. We further identified heart disease (ICD-9 codes 391-429 or ICD-10 codes I01-I51), acute myocardial infarction (ICD-9 codes 410-414, ICD-10 codes I21-I22) and stroke (ICD-9 codes 430-438, ICD-10 codes I60-I69) from the cardiovascular disease category [16].

We obtained air pollution and meteorological data from the Environmental Protection Agency, USA (USA Air Quality System: http://www.epa.gov/ttn/airs/airsaqs/detaildata/downloadaqsdata.htm). Hourly concentrations of ambient ozone were recorded at each of eight stationary monitoring sites located in these counties set up by the agency. We first calculated means of hourly ozone concentration from all eight monitoring sites and then calculated daily maximal 8-hour concentrations during the study period. The moving averages of daily maximal 8-hour ozone up to seven days were used as personal exposure. We used maximal 8-hour concentration of ambient ozone because WHO recommended that maximal 8-hour mean reflects the most health-related exposure to ozone [23]. We used apparent temperature as an index, defined as a person's perceived air temperature, given the humidity [24].

### Data analysis

The case-crossover design was applied to estimate the association between ozone and mortality. This is a special case-control design when each subject is matched to himself or herself. Controls are chosen as defined days when the subject does not experience the study outcomes. Since each case-set is made of a single subject, all individual time-invariant characteristics are perfectly matched, and therefore, each potential time-invariant known or unknown individual confounder is automatically adjusted for. There are several referent selection strategies in case-crossover designs [25]. Referent days in this study were selected according to the time-stratified approach, by dividing the study period into monthly strata. We selected every third day within the stratum of the case event as referents of the event to decrease serial correlation and excluded referents within seven days before and after the case event because we used up to seven day-moving averages of ozone as exposure indices and the accumulative effects of ozone effect might offset estimations. We then fit the conditional logistical regression models to estimate associations between ozone exposure and mortality and examine effect modification using PROC PHREG in SAS (SAS software Release 9.1; SAS Institute, Cary, NC, 20084) [25].

Because days of the week effect were not explicitly adjusted for in our study design, we adjusted for days of week as a categorical variable in each model. We used a moving average of daily apparent temperature and its quadratic term in each model because of potential nonlinear relationships between apparent temperature and health outcomes. In order to determine the period of moving averages of the temperature, we separately fit models that only included linear and quadratic terms of a moving average of temperature and days of the week and replaced the temperature terms with different day-moving averages of temperature from the current day to seven days. The four-day moving average of daily apparent temperature was chosen in the final models to minimize the Akaike information criterion (AIC) index. We then estimated the effects of ozone on health outcomes adjusting for temperature and days of the week.

To examine the effect modification by each selected variable or modifier (a categorical variable), we separately fit models including the interactive term for ozone and the modifier at a time. We then linearly combined the coefficients for referent group and interactive terms to assess the associations by the modifier. We were particularly interested in the impacts of a subject specific measure of socioeconomic status (educational attainment) and area based measures (median household income, poverty). All analyses were restricted to the periods between May 1st and September 30th, 1995-2002 because the concentrations of ozone in cold seasons were very low in the area and many other studies only examined ozone effects in warmer seasons, or found such an association only existed in the warm seasons [1,2,8,9,26,27].

## Results

Table 1 shows the demographic description for the decedents. Results show that nearly 65% of deaths were over 75 years old and white was the dominant race, but 16.51% died before 65 years old for the whole period. 64.3% of the decedents had between 9 and12 years of education and over one quarter had 13 years education or more. Females were composed of 55.62% of the deaths. Averages of maximal 8-hour, daily ozone and daily apparent temperature were 41.46 (Standard Deviation (SD):17.26) ppb, 27.51 (SD: 12.42) ppb, and 18.32 (SD: 6.97) °C during May-September, 1995-2002, respectively.

**Table 1:** Demographical statistics of study population (deaths = 157,197) in Eastern Massachusetts, 1995-2002

Variable	No. (%) of the whole period Subjects = 157,197	No. (%) between May-Sept Subjects = 60,302
Age		
35-64	25958 (16.51)	10439 (17.31)
65-74	29619 (18.84)	11709 (19.42)
75-84	48757 (31.02)	18581 (30.81)
85 or older	52863 (33.63)	19573 (32.46)
Education		
≤ 8 years	13516 (8.60)	5151 (8.54)
9-12	101083 (64.30)	38729 (64.23)
13-16	30888 (19.65)	11929 (19.78)
17 or more	9844 (6.26)	3774 (6.26)
Unknown	1866 (1.19)	719 (1.19)
Marital		
Never married	22051 (14.03)	5151 (13.79)
Married or separated	56203 (35.75)	38729 (36.54)
Widowed	66291 (42.17)	11929 (41.38)
Divorced	12383 (7.88)	3774 (8.12)
Unknown	269 (0.17)	112 (0.19)
Cause of death		
CVD	59354 (37.76)	22585 (37.45)
MI	20705 (13.17)	7853 (13.02)
Stroke	1019 (6.18)	3883 (6.44)
Other heart	28458 (18.10)	10849 (17.99)
Respiratory disease	16751 (10.66)	5697 (9.45)
Diabetes	3845 (2.45)	1524 (2.53)
All Other	77247 (49.14)	30496 (50.57)
Gender		
Male	69758 (44.38)	27052 (44.86)
Female	87439 (55.62)	33250 (55.14)
Race		
White & non-Hispanic	144646 (92.02)	55348 (91.78)
Black	8381 (5.33)	3330 (5.52)
American Indian	54 (0.03)	23 (0.04)
Hispanic	1289 (0.82)	509 (0.84)
Asian-Pacific	2184 (1.39)	849 (1.41)
Others	12 (0.01)	4 (0.01)
Unkown	631 (0.40)	239 (0.40)

We separately assessed associations using ozone on the current day to moving averages up to the previous week. All presented results show effects of ozone on all natural deaths after adjusting for days of the week and linear and quadratic terms for the four-day moving average of apparent temperature (Table 2). The estimated associations show little change across different moving averages. Because the estimate was slightly stronger than those for other moving-averages, we used the four-day moving average in the analysis of specific causes of death and effect modifications. Per 10 ppb increase of the average of maximal 8-hour ozone in the previous week was associated with 1.61% (95% confidence interval (CI): 0.25%, 3.00%) increase in non-accident deaths. The interquartile ranges (IQR) of ozone were varied across different daily moving days and longer moving day, the lower concentration.

**Table 2:** Estimated percent increase in natural mortality per 10 ppb in average of maximal 8-hour ozone

	IQR (ppb)	% increase (95% CI)
Current day	20.79	1.32 (0.74, 1.91)
Moving average at 1-day lag	19.11	1.66 (0.96, 2.36)
Moving average at 2-day lag	16.75	1.61 (0.78, 2.45)
Moving average at 3-day lag	15.47	1.61 (0.61, 2.62)
Moving average at 4-day lag	14.78	1.68 (0.51, 2.87)
Moving average at 5-day lag	13.96	1.56 (0.27, 2.87)
Moving average at 6-day lag	13.45	1.61 (0.25, 3.00)

Adjusting for linear and quadratic four-day moving average of temperature and days of week among those aged 35 or older in Eastern Massachusetts, May-September, 1995-2002. CI: confidence interval; IQR: interquartile range.

**Figure 1:**
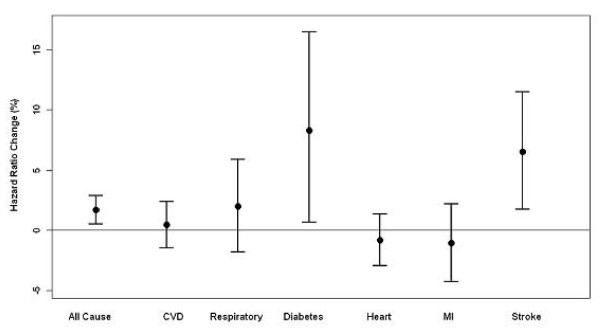
**Percent increases in mortality per 10 ppb increase in 4-day averages of maximal 8-hour ozone**. Adjusting for linear and quadratic apparent temperature and days of week among adults aged 35 years or older in the Eastern Massachusetts, May-September, 1995-2002. The circles mean point estimates and bars refer to 95% confidence intervals. CVD: Cardiovascular diseases; MI: Myocardial infarction; circles refer to point estimates and bars refer to 95% confidence intervals.

We further examined associations of exposure to the four-day moving average of maximal 8-hour ozone with specific causes of death, including cardiovascular diseases, diabetes, respiratory disease, heart diseases, acute myocardial infarction and stroke, adjusting for days of the week and apparent temperature as above. Figure 1 shows that exposure to ozone was strongly associated with diabetes and stroke, slightly stronger with respiratory disorders, but weakly with heart diseases, myocardial infarction or cardiovascular diseases. Per 10 ppb increase in the average of maximal 8-hour ozone in the previous four days was associated with 1.96% (95% CI: -1.83%, 5.90%), 8.28% (95% CI: 0.66%, 16.48%), 0.44% (95% CI: -1.45%, 2.37%), -0.83% (95% CI: -2.94%, 1.32%), -1.09% (95% CI: -4.27%, 2.19%) and 6.50% (95% CI: 1.74%, 11.49%) changes in deaths from respiratory disorders, diabetes, cardiovascular diseases, heart, acute myocardial infarction and stroke, respectively.

We then separately examined whether or not each of socioeconomic status and individual characteristics modified the association between non-accident deaths and the four-day moving average of maximal 8-hour ozone. Table 3 shows those results treating each potential modifier one by one. This study did not find any obvious evidence that the association between ozone and non-accident mortality was modified by age, gender, race, education, marital status, population density, median household income and percent poverty in the block group (all p-values of coefficients of interaction terms were more than 0.2). Of note, we found little evidence that ozone only affected the elderly, with a significant association with mortality in the 35-64 year age group that was essentially identical in size to that for the oldest age group. Interactions with educational level and median household income were likewise not significant. We also examined effect modifications for other outcomes including respiratory disorders, CVD and stroke. As non-accident deaths, again, there was no obvious evidence demonstrating effect modifications (Table 4).

**Table 3:** Percent changes in natural deaths per 10 ppb of four-day average of maximal 8-hour ozone

Variable	% increase (95% CI)
Age	
35-64 years	1.48 (0.28, 2.70)
65-74 years	1.54 (0.34, 2.75)
75-84 years	1.59 (0.42, 2.77)
≥ 85 years	1.51 (0.34, 2.70)
Education	
≤ 8 years	1.53 (0.26, 2.83)
9-12 years	1.54 (0.39, 2.70)
13-16 year	1.56 (0.36, 2.77)
17 years or more	1.46 (0.13, 2.80)
Marital status	
Never Married	1.54 (0.32, 2.78)
Married or separated	1.51 (0.34, 2.69)
Widowed	1.57 (0.41, 2.74)
Divorced	1.49 (0.21, 2.79)
Race	
White & non Hispanic	1.54 (0.39,2.70)
Black	1.47 (0.12,2.84)
Indian	1.26 (-7.46,10.81)
Hispanic	1.33 (-0.87,3.59)
Asian or Pacific	1.63 (-0.21,3.50)
Others	0.93 (-19.24,26.15)
Hispanic	
Hispanic	1.54 (0.39,2.69)
Non Hispanic	1.53 (-0.43,3.52)
White	
White	1.54 (0.39,2.69)
Non white	1.52 (0.23,2.83)
Gender	
Male	1.55 (0.39,2.72)
Female	1.53 (0.37,2.69)
Population per km^2 ^*	
Low	1.5 (0.32,2.69)
Medium	1.54 (0.38,2.71)
High	1.57 (0.38,2.78)
Household income*	
Low	1.58 (0.39,2.79)
Medium	1.51 (0.36,2.68)
High	1.56 (0.37,2.76)
Percent poverty*	
Low	1.47 (0.3,2.66)
Medium	1.55 (0.4,2.72)
High	1.58 (0.37,2.8)

Adjusting for linear and quadratic apparent temperature and days of week in the Eastern Massachusetts, May-September, 1995-2002. CI: confidence interval; * Use low and high quartiles as cut-offs. Low and high quartiles are 1016.35 and 6400 residents per km^2 ^for population density, US$47,264 and US$84,047 for household income, 3.42% and 14.64% for percent poverty, respectively.

**Table 4:** Percent changes in specific cause deaths per 10 ppb of four-day average of 8-hour ozone

Variable	CVD	Respiratory Disorders	Diabetes
Age			
35-64 years	0.7 (-1.29,2.73)	1.62 (-2.65,6.08)	8.95 (0.82,17.73)
65-74 years	0.77 (-1.19,2.76)	1.73 (-2.21,5.82)	8.03 (0.13,16.56)
75-84 years	0.66 (-1.23,2.59)	2.1 (-1.71,6.06)	8.05 (0.32,16.39)
≥ 85 years	0.61 (-1.26,2.53)	2.13 (-1.64,6.04)	6.79 (-1.05,15.24)
Education			
≤ 8 years	0.6 (-1.44,2.69)	1.99 (-2.16,6.32)	7.36 (-1.52,17.03)
9-12 years	0.65 (-1.21,2.54)	2.01 (-1.71,5.88)	7.73 (0.29,15.72)
13-16 year	0.74 (-1.19,2.72)	1.91 (-2.02,6)	8.56 (0.33,17.47)
17 years or more	0.72 (-1.47,2.96)	2.06 (-2.49,6.83)	9.01 (-1.4,20.51)
Marital			
Never Married	0.71 (-1.27,2.72)	2.21 (-1.79,6.37)	8.55 (0.2,17.59)
Married or separated	0.63 (-1.26,2.55)	1.8 (-2.02,5.77)	8.18 (0.52,16.42)
Widowed	0.67 (-1.2,2.57)	2.08 (-1.68,5.98)	7.46 (-0.17,15.67)
Divorced	0.75 (-1.37,2.91)	1.92 (-2.36,6.39)	7.6 (-1.5,17.53)
Race			
White & non-Hispanic	0.66 (-1.18,2.54)	2.04 (-1.67,5.88)	8.39 (0.97,16.37)
Black	0.6 (-1.6,2.85)	1.59 (-3.45,6.9)	7.05 (-1.71,16.59)
Indian	0.3 (-14.7,17.93)	4.88 (-23.3,43.42)	-92.17 (-99.78,174.62)
Hispanic	1.08 (-2.88,5.2)	0.85 (-8.16,10.75)	5.25 (-7.77,20.11)
Asian or Pacific	0.41 (-2.8,3.72)	1.42 (-5.04,8.31)	11.36 (-2.44,27.11)
Hispanic			
Hispanic	0.66 (-1.19,2.54)	2.02 (-1.68,5.86)	7.99 (0.61,15.91)
Non Hispanic	1.3 (-2.12,4.85)	1.25 (-6.56,9.71)	5.22 (-6.87,18.86)
White			
White	0.66 (-1.18,2.55)	2.04 (-1.66,5.88)	8.02 (0.62,15.96)
Non white	0.65 (-1.47,2.81)	1.48 (-3.08,6.27)	7.17 (-1.23,16.28)
Gender			
Male	0.68 (-1.2,2.58)	2.16 (-1.62,6.08)	8.23 (0.66,16.36)
Female	0.65 (-1.21,2.55)	1.9 (-1.83,5.78)	7.62 (0.12,15.69)
Population per km^2 ^*			
Low	0.65 (-1.25,2.59)	2.18 (-1.66,6.16)	8.2 (0.3,16.73)
Medium	0.65 (-1.21,2.55)	1.93 (-1.82,5.81)	8.02 (0.5,16.11)
High	0.71 (-1.22,2.68)	2 (-1.91,6.07)	7.39 (-0.45,15.86)
House income*			
Low	0.69 (-1.24,2.66)	1.95 (-1.97,6.02)	7.56 (-0.3,16.05)
Medium	0.61 (-1.25,2.5)	2.05 (-1.69,5.93)	7.91 (0.42,15.96)
High	0.78 (-1.14,2.74)	1.96 (-1.91,5.99)	8.39 (0.17,17.3)
Percent poverty*			
Low	0.61 (-1.29,2.55)	1.92 (-1.92,5.9)	7.72 (-0.14,16.2)
Medium	0.68 (-1.18,2.58)	2.08 (-1.66,5.96)	8.12 (0.59,16.21)
High	0.68 (-1.28,2.67)	1.93 (-2.05,6.06)	7.64 (-0.32,16.23)
Age			
35-64 years	-0.48 (-2.68,1.78)	0.06 (-3.35,3.58)	6.15 (0.8,11.78)
65-74 years	-0.45 (-2.63,1.77)	0.23 (-3.06,3.64)	6.36 (1.21,11.78)
75-84 years	-0.5 (-2.62,1.66)	0.03 (-3.17,3.34)	5.85 (1.07,10.86)
≥ 85 years	-0.58 (-2.68,1.56)	-0.03 (-3.23,3.27)	5.93 (1.21,10.87)
Education			
≤ 8 years	-0.62 (-2.9,1.71)	-0.07 (-3.54,3.53)	6.15 (0.86,11.71)
9-12 years	-0.52 (-2.59,1.6)	0.07 (-3.08,3.32)	5.79 (1.12,10.68)
13-16 year	-0.47 (-2.63,1.74)	0.06 (-3.24,3.47)	6.41 (1.49,11.57)
17 years or more	-0.44 (-2.89,2.07)	0.17 (-3.62,4.12)	6.15 (0.45,12.18)
Marital			
Never Married	-0.39 (-2.59,1.86)	-0.05 (-3.45,3.46)	5.77 (0.65,11.14)
Married or separated	-0.58 (-2.68,1.57)	0.06 (-3.13,3.36)	5.97 (1.17,10.99)
Widowed	-0.53 (-2.62,1.6)	0.05 (-3.13,3.33)	6.01 (1.3,10.94)
Divorced	-0.43 (-2.78,1.97)	0.21 (-3.47,4.03)	6.17 (0.37,12.31)
Race			
White & non Hispanic	-0.51 (-2.57,1.6)	0.07 (-3.06,3.3)	5.92 (1.27,10.77)
Black	-0.71 (-3.16,1.79)	-0.26 (-4.18,3.82)	6.75 (0.82,13.03)
Indian	0.12 (-17.54,21.57)	0.14 (-21.52,27.78)	-0.49 (-37.63,58.76)
Hispanic	-0.64 (-5.11,4.04)	-0.7 (-8.16,7.38)	9.06 (-1.32,20.53)
Asian or Pacific	-0.83 (-4.54,3.02)	0.14 (-5.82,6.48)	5.4 (-2.42,13.85)
Hispanic			
Hispanic	-0.52 (-2.58,1.58)	0.05 (-3.07,3.28)	5.95 (1.31,10.8)
Non Hispanic	-0.28 (-4.19,3.8)	-0.44 (-6.95,6.52)	8.3 (-0.35,17.71)
White			
White	-0.51 (-2.57,1.59)	0.06 (-3.07,3.3)	5.92 (1.28,10.77)
Non white	-0.69 (-3.05,1.73)	-0.2 (-3.93,3.68)	6.72 (1.17,12.57)
Gender			
Male	-0.51 (-2.6,1.62)	0.1 (-3.08,3.37)	6.1 (1.33,11.1)
Female	-0.53 (-2.61,1.59)	0.01 (-3.15,3.27)	5.9 (1.23,10.8)
Population per km^2 ^*			
Low	-0.46 (-2.59,1.71)	-0.02 (-3.25,3.32)	5.56 (0.75,10.59)
Medium	-0.58 (-2.66,1.54)	-0.01 (-3.17,3.25)	6.24 (1.53,11.17)
High	-0.44 (-2.6,1.76)	0.31 (-2.99,3.72)	5.81 (0.87,11)
House income*			
Low	-0.53 (-2.69,1.67)	0.17 (-3.11,3.58)	6.11 (1.14,11.32)
Medium	-0.55 (-2.63,1.56)	0 (-3.15,3.26)	5.77 (1.09,10.67)
High	-0.42 (-2.57,1.77)	0.07 (-3.21,3.46)	6.35 (1.48,11.44)
Percent poverty*			
Low	-0.61 (-2.73,1.56)	-0.15 (-3.37,3.19)	6.16 (1.35,11.2)
Medium	-0.46 (-2.54,1.66)	0.16 (-3,3.42)	5.75 (1.06,10.66)
High	-0.58 (-2.76,1.64)	-0.03 (-3.38,3.43)	6.41 (1.37,11.7)

Adjusting for linear and quadratic apparent temperature and days of week in the Eastern Massachusetts, May-September, 1995-2002.CI: confidence interval; CVD: Cardiovascular Disease; * Use low and high quartiles as cut-offs.

## Discussion

In this study, exposure to ambient ozone in the short term was positively associated with daily deaths in Eastern Massachusetts during May-September, 1995-2002, consistent with evidence from other parts of the world [1,21]. We saw no consistent gradients in effect modification by individual characteristics and social economic status, and in particular no gradient by either individual level socioeconomic status or area-based socioeconomic status, which was based on small areas (block groups) that were designed to minimize within area gradients while maximizing between area gradients. We did find that the effect was seen in the 35-64 year age group, with essentially the same risk as for older ages, indicating that the effects of ozone are not limited to persons with little life expectancy remaining. In addition, we found that the estimates were stronger when the average periods were extended.

There is a substantial epidemiological literature showing clear and consistent association between exposure to ambient ozone and mortality [5,9,13,22]. Of great interest are biological mechanisms by which ambient ozone could cause morbidity and mortality. One way investigators can identify the potential mechanisms is to assess whether some subpopulations with specific characteristics or at different socioeconomic status are especially sensitive to health effects. The identification of sets of individuals who are more sensitive to exposure to ozone may suggest possible mechanisms of physiological attacks as well as provide clues for more detailed risk assessment. However, it should be noted that the vulnerability may also indicate other non biological factors, such as health care access issues.

Several other studies also examined different vulnerability between specific causes when they examined associations between ozone air pollution and human health, but findings were inconsistent [2,27]. Gryparis et al. conducted a time-series study to investigate associations between ozone and mortality in 21 European cities and found that a 10-ppb increase in the 2-day average of 8-hour ozone was associated with 0.31% (95% CI: 0.17%, 0.52%), 1.13% (95% CI: 0.74%, 1.51%) and 0.46% (95% CI: 0.22%, 0.73%) increases in total mortality, respiratory and cardiovascular mortality in summer, respectively [28]. Bell et al. conducted a meta-analysis of 144 effect estimates from 39 time-series studies and found that a 10-ppb increase of ozone at short-term lags were related to 0.87% (95% posterior interval or PI: 0.55%, 1.18%), 0.47% (95% PI: -0.51%, 1.47%) and 1.11% (95% PI: 0.68%, 1.53%) increases in total, respiratory and cardiovascular mortality, respectively [2]. The two large multi-city time-series studies found that these vulnerabilities of respiratory and cardiovascular diseases to exposure to ozone were controversial.

So far, a few studies examined the vulnerability of subpopulations with diabetes or stroke to ozone air pollution [21]. Medina-Ramün and Schwartz [21] examined who were particularly vulnerable to ozone air pollution in 48 US cities and did not find that persons who died from stroke or diabetes were more susceptible to ozone air pollution compared to those who died from other specific causes. Our data suggest that people with diabetes or stroke were at more risk than people with more general heart or lung conditions. A 10-ppb increase in a four-day moving average of maximal 8-hour ozone was associated with 1.68% (95% CI: 0.51%, 2.87%), 1.96% (95% CI: -1.83%, 5.90%) and 0.44% (95% CI: -1.45%, 2.37%) increases of total, respiratory and cardiovascular mortality, respectively. This issue remains clarified for other studies.

Some studies examined such vulnerabilities for particulate air pollution and found that people with diabetes were more sensitive to particulate matter pollution [13,16,19,29,30]. For example, Zanobetti et al. found that the concurrent diabetes doubled the risk of particulate matter to cardiovascular disease [29]. Goldberg et al found that those with diabetes were more susceptible to particles [30]. Zeka et al. reported that a secondary diagnosis of diabetes modified the associations of PM_10 _with respiratory and stroke mortality [16]. O'Neill et al. examined vulnerability among 270 greater-Boston residents and found that persons with diabetes were more susceptible to particulate air pollution [19].

Recently, a small number of studies assessed the effect modifications of ambient ozone on health by individual characteristics and socioeconomic status [21,22]. Medina-Ramün & Schwartz reported that in a multiple community study, each 10 ppb ozone increase (average 0-2 lags) was associated with 1.10% additional increase in mortality of people aged ≥ 65 years compared with younger age [21]. They also reported that women over age 60 and black people were particularly susceptible. Bell & Dominici examined the effect modification of ozone and mortality in 98 US communities by socioeconomic statuses at a city level [22]. They found that community-level factors modified the association between ambient ozone and mortality. They stated that higher effect estimates were associated with higher unemployment and the fraction of the Black/Africa-American population. However, this study did not find any evidence that individual characteristics and socioeconomic levels modified associations between ozone and mortality. In contrast, we find the people aged 35-64 experienced similar risk of death due to ozone exposure, which was different from findings from other studies [1,21,31-33].

There are several possible explanations for this divergence. Given a limited area, our findings could be by chance. On the other hand, the Bell and Dominici's results may reflect the impact of regional differences in socioeconomic status and racial composition, which could be standing for different things than the within urban differences we examined. For example, diet and lifestyle differences could show different correlations with such variables between *vs *within city. The differences between this assessment of individual level effect modifiers and the paper of Medina-Ramon et al. [4] is more likely due to the much smaller number of deaths examined. We were not able to look at gender differences stratified by age, as in that paper, for example. The differences may also be attributable to the different methodology used at some extent.

Although many previous studies recently reported effect modification of exposure to ambient air pollutants by socioeconomic statuses, individual characteristics, weather conditions or even genotypes, a very small proportion of them directly examined whether or not estimated effect modifications are significant [8-10,17]. Some studies examined effect modifications at community levels at the second-stage meta-analyses [22,34], but this design could not efficiently examine whether there is a significant effect modification at an individual level. The estimated effect modifications could be partially attributable to differential bias from exposure misclassification caused by some factors such as temperature, age, education and poverty because these factors are related to residential behaviors in communities. Similarly, in the stratification studies dividing a whole population into subpopulations based on a factor [12,20], the different estimated effects across subpopulations might be interpreted to some extent as differential distributions of the risk factors adjusted or unadjusted for in subpopulations because subpopulations were not exchangeable for other factors. Moreover, these designs did not directly examine an interactive effect but simply main effects in terms of statistical theory [35,36]. As we know, in any epidemiological studies, estimated associations are subject to true risk, variations attributable to differential distributions of many factors adjusted or unadjusted for across populations, bias from error measures of dependent and independent variables as well as a chance. Therefore, the different effect estimates across populations or subpopulations would be commonly expected. If inconsiderable different estimates between subpopulations or populations are demonstrated in these kinds of study designs, the interpretation of the differences as an effect modification should be particularly cautious.

Another important issue may be publication bias [37-42]. Journals tend to publish papers reporting significant findings and those without significant findings are less likely to be published even though their study designs were not poorer or even better than those published. Similarly, authors tend to report significant results and the uncertainty of model selections helps significant findings. Although the issue has been criticized by many authors, papers reporting non-significant findings may still be discriminated to some extent because journals tend to pursue high citations [42].

This study used geographic information system (GIS) (ArcGIS, ESRI, Redlands, CA) to identify the geographic coordinates of personal residential addresses and then link the addresses to the census tract dataset to obtain socioeconomic status information. This study used a case-crossover design to identify effect modification at individual levels and test the significance of interaction. All time-invariant variables were autonomically controlled by design except for temperature and days of the week. Therefore, the study design has its own advantages. However, as in many air pollution studies, the average of ozone measures at the several fixed monitoring sites as a surrogate of individual exposures might result in misclassification bias. The extent of error depends on the spatial homogeneity of the exposure, as we compared exposures with temporality. A recent study compared ambient concentrations with personal exposures with monitoring measurement and results showed high correlation between these two measurements for PM_2.5_in the Boston area [43]. Although there is no exact evidence for the homogeneity of ozone in this study, in general, the gaseous pollution is more homogeneous than particulate air pollution. Nevertheless, if the misclassification is nondifferential, any potential bias will tend toward the null. In addition, this study only included three counties in the eastern Massachusetts with over 1,570,000 of population. The limited population and the local area restricted its generalization to other places.

## Conclusions

This study found consistent associations between exposure to ambient ozone and mortality (all natural deaths, respiratory disorders, CVD and stroke) adjusting for apparent temperature and days of week. There was no evidence that the individual characteristics and socioeconomic statuses (age, race, gender, education, marital status, local population density, average household income and percent poverty at the census blocks) modified associations between exposure to maximal 8-hour ozone and natural or cause-specific mortality in previous four days.

## Abbreviations

SES: socioeconomic status; ICD: International Classification of Disease; CVD: cardiovascular diseases; AIC: Akaike information criterion; SD: Standard Deviation; IQR: interquartile ranges; GIS: geographic information system

## Competing interests

The authors declare that they have no competing interests.

## Authors' contributions

CR was responsible for study design, data analyses, result interpretation and manuscript writing. SM was responsible for gecoding of data, socioeconomic data collection and result interpretation. JS was responsible for study design, data collection and result interpretation. All authors read and approved the final manuscripts.
